# Deep learning-based approaches for multi-omics data integration and analysis

**DOI:** 10.1186/s13040-024-00391-z

**Published:** 2024-10-02

**Authors:** Jenna L. Ballard, Zexuan Wang, Wenrui Li, Li Shen, Qi Long

**Affiliations:** 1 Graduate Group in Genomics and Computational Biology, Perelman School of Medicine, University of Pennsylvania, 3700 Hamilton Walk, Philadelphia, PA 19104 USA; 2 Graduate Group in Applied Mathematics and Computational Science, University of Pennsylvania, 209 S. 33rd Street, Philadelphia, PA 19104 USA; 3 Department of Statistics, University of Connecticut, 215 Glenbrook Road, Storrs, CT 06269 USA; 4 Department of Biostatistics, Epidemiology and Informatics, Perelman School of Medicine, University of Pennsylvania, 423 Guardian Drive, Philadelphia, PA 19104 USA

**Keywords:** Deep learning, Generative model, Multi-omics integration, Imaging

## Abstract

**Background:**

The rapid growth of deep learning, as well as the vast and ever-growing amount of available data, have provided ample opportunity for advances in fusion and analysis of complex and heterogeneous data types. Different data modalities provide complementary information that can be leveraged to gain a more complete understanding of each subject. In the biomedical domain, multi-omics data includes molecular (genomics, transcriptomics, proteomics, epigenomics, metabolomics, etc.) and imaging (radiomics, pathomics) modalities which, when combined, have the potential to improve performance on prediction, classification, clustering and other tasks. Deep learning encompasses a wide variety of methods, each of which have certain strengths and weaknesses for multi-omics integration.

**Method:**

In this review, we categorize recent deep learning-based approaches by their basic architectures and discuss their unique capabilities in relation to one another. We also discuss some emerging themes advancing the field of multi-omics integration.

**Results:**

Deep learning-based multi-omics integration methods were categorized broadly into non-generative (feedforward neural networks, graph convolutional neural networks, and autoencoders) and generative (variational methods, generative adversarial models, and a generative pretrained model). Generative methods have the advantage of being able to impose constraints on the shared representations to enforce certain properties or incorporate prior knowledge. They can also be used to generate or impute missing modalities. Recent advances achieved by these methods include the ability to handle incomplete data as well as going beyond the traditional molecular omics data types to integrate other modalities such as imaging data.

**Conclusion:**

We expect to see further growth in methods that can handle missingness, as this is a common challenge in working with complex and heterogeneous data. Additionally, methods that integrate more data types are expected to improve performance on downstream tasks by capturing a comprehensive view of each sample.

## Background

Exploring the biological mechanisms of human health is a core aspect of biomedical research. The advent of high-throughput technologies has significantly broadened our ability to analyze the biological underpinnings of life at various levels of complexity. Multiomics, or integrative omics or panomics, is a comprehensive approach to biological analysis. It involves simultaneously studying multiple ‘omics’ datasets, including the genome, proteome, transcriptome, epigenome, metabolome, and microbiome. This approach allows researchers to explore the complex interactions and networks underlying biological processes and diseases.

Many studies have demonstrated that multi-omics data can offer valuable insights into understanding biological processes. Bakker et al. [1] shows that by integrating multi-omics layers, cytokine production is influenced by various genetic and non-genetic factors and can be moderately predicted using baseline profiles. Nativio et al. [2] conducted a comprehensive multi-omics analysis of brains affected by Alzheimer’s disease (AD) compared to those of older and younger controls. Their study identified histone modifications associated with AD and revealed that increases in H3K27ac and H3K9ac in AD brains disrupt disease pathways by affecting transcription and chromatin-gene feedback loops. Zijlmans et al. [3] utilized an integrated multi-omics approach to map the chromatin-associated proteome, histone post-translational modifications (hPTMs), and transcriptome of naive and primed human pluripotent stem cells (hPSCs). They unexpectedly discovered that PRC2 activity inhibits trophoblast induction in naive hPSCs and blastoids, revealing that naive pluripotent cells are not epigenetically unrestricted but are constrained in their differentiation into trophoblast by chromatin barriers.

Multi-omics provides a comprehensive approach that enhances discovery across various biological levels. However, it faces several challenges in practice: (1) Paired and Unpaired Datasets - Ideally, these studies should use paired samples, where all omics layers per replicate are derived from a single individual. Two issues arise with unpaired samples: different sample sources and data modalities. Different sample sources refer to collecting omics data from distinct batches of cells or biological samples. Conversely, different data modalities refer to the simultaneous sequencing of various types of omics data from the same sample set. Analytical methods such as correlation analysis detect relationships between various omics layers across the dataset [4]. Deep learning methods are employed for differing data modalities to transform the data into the shared latent space by autoencoders and then perform integration [5]. (2) Missing Values - Common in multi-omics datasets, missing values can result from experimental limitations or sample quality issues. Bayesian methods and deep learning-based methods are often used to address this problem. (3) High dimensionality - Multi-omics datasets often encompass thousands of genes, leading to a high-dimensional data space. This can pose challenges in data analysis, as traditional statistical methods may struggle with the “curse of dimensionality”. Dimensionality reduction techniques such as principal component analysis (PCA) [6], t-distributed stochastic neighbor embedding (t-SNE) [7], and uniform manifold approximation and projection (UMAP) are commonly employed [8].

Different approaches have been developed to address the practical issues in multi-omics data integration, and these can be categorized into statistical learning methods and machine learning-based approaches. Among the statistical learning methods, a famous one is Principal Component Analysis (PCA) and its variants, which aim to reduce the dimensionality of the data while preserving as much of the variance as possible. A method similar to PCA is Canonical Correlation Analysis (CCA), which seeks to find linear combinations of variables in two datasets that are maximally correlated. In traditional machine learning algorithms, Multi-kernel frameworks are often used to integrate multiple datasets of various types into a single exploratory analysis [9]. Deep learning, a branch of machine learning, is increasingly popular for its capability to identify complex nonlinear patterns in data. It offers an efficient framework for processing large volumes of multi-omics data and has a strong generalization capacity, which allows it to make accurate predictions for unseen data [10].

There are several reviews about multi-omics data integration. Subramanian et al. [11] summarized Multi-omics Data integration tasks based on the traditional machine learning algorithm and classified them into network, Bayesian, fusion, similarity-based, correlation-based, and other multivariate methods but did not discuss the deep learning side. Vahabi and Michailidis [12] reviewed the method for unsupervised learning tasks in Multi-Omics Data Integration. Wekesa and Kimwele [13] explored the application of deep learning in disease diagnosis, prognosis, and therapies in multi-omics data integration but is only limited to convolutional neural networks (CNN), feed-forward networks, and recurrent neural networks (RNN). Kang et al. [14] presented a review of recent deep learning-based studies that integrate multi-omics data for downstream analysis, including feature selection/reduction, clinical outcome prediction, survival analysis, and clustering for subtype discovery. Wen et al. [15] reviewed the multi-omics data integration methods based on the different DL frameworks: fully connected neural network (FCNN), convolutional neural network (CNN), autoencoder (AE), graph neural network (GNN), capsule network (CapsNet), and generative adversarial network (GAN).

**Fig. 1:**
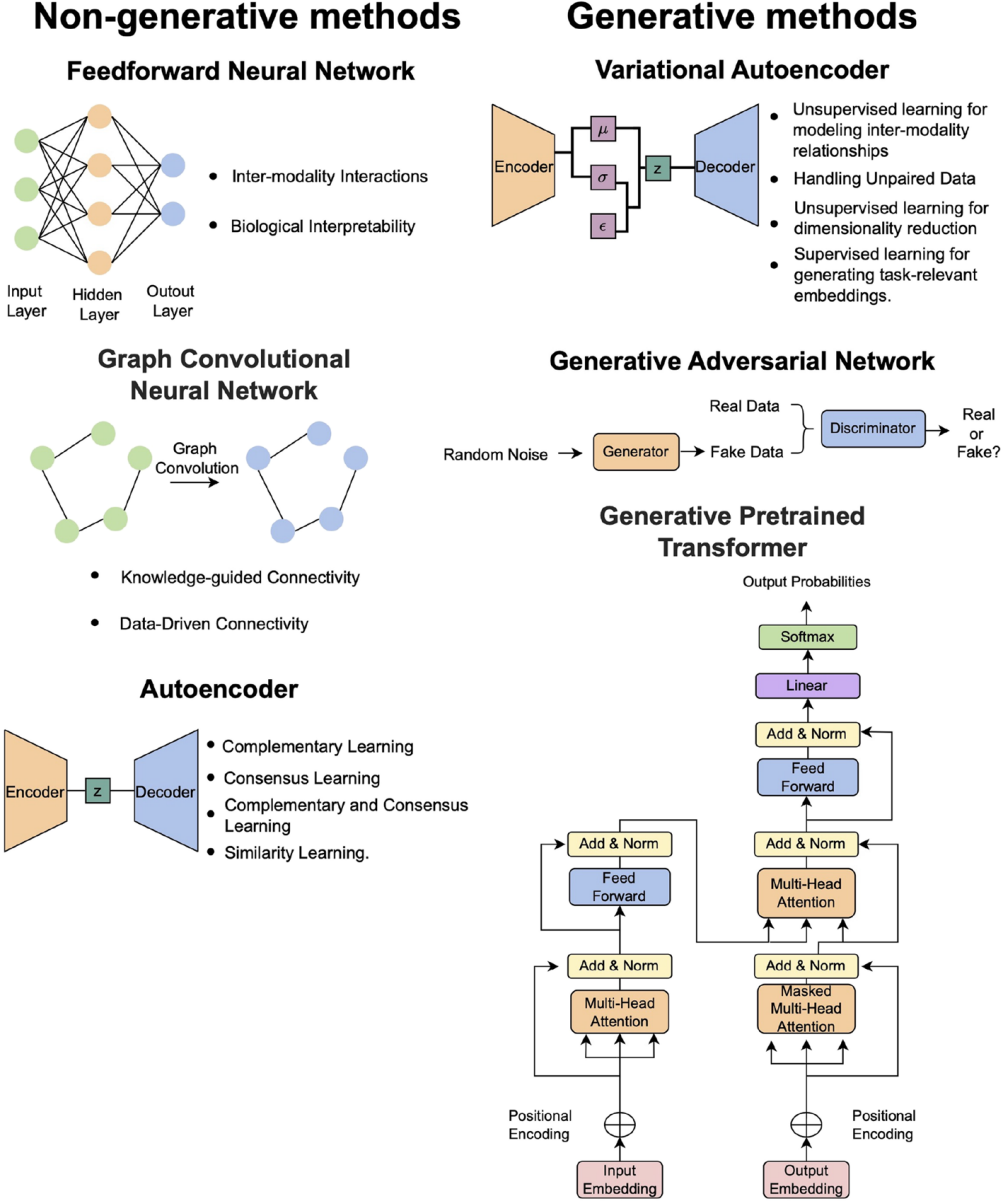
Overview of the types of methods reviewed in this paper

**Fig. 2:**
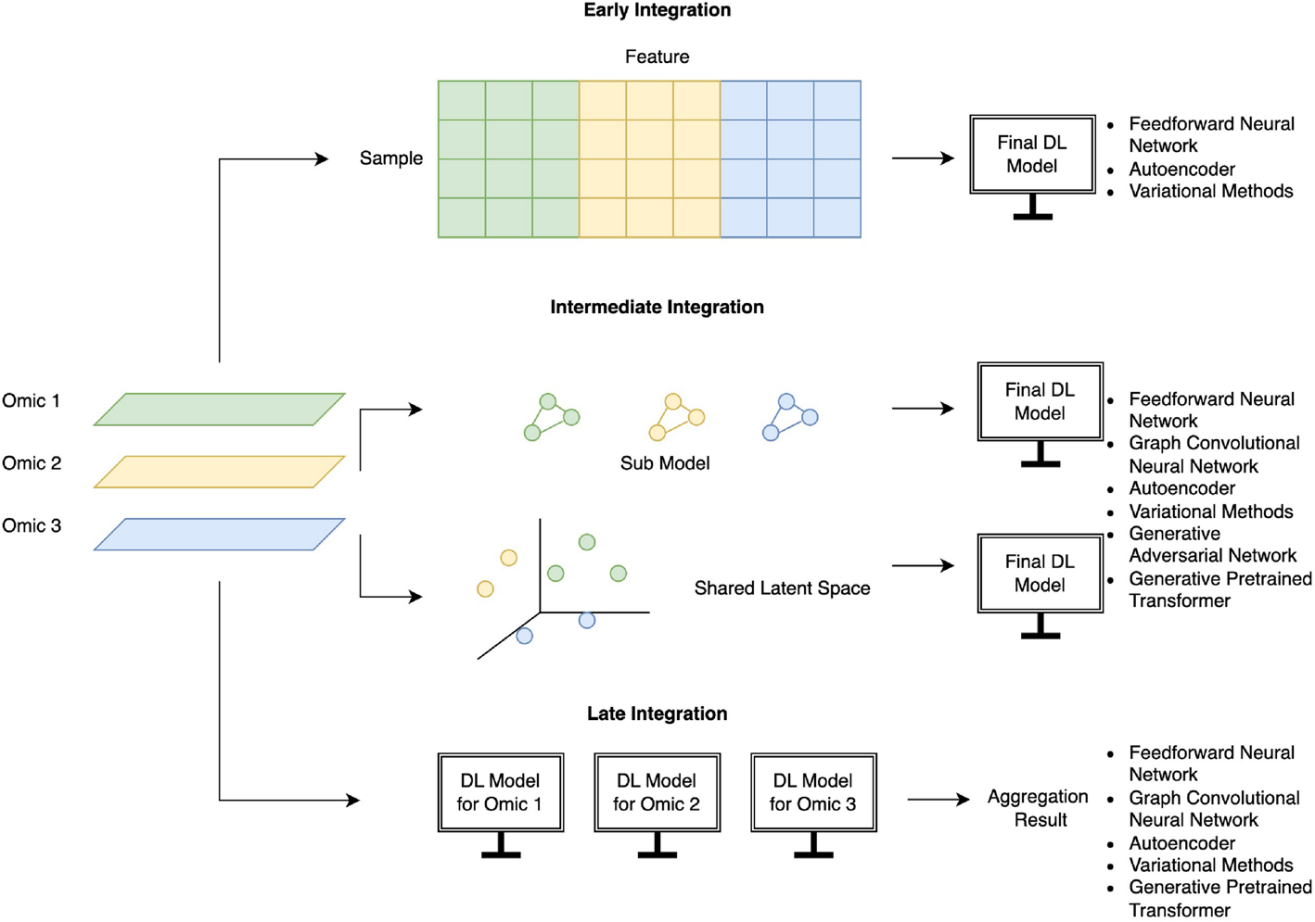
Overview of multi-modal integration strategies

This review focuses on the tools and methods published since 2017 that integrate multiple omics data and discusses their applications in understanding complex human biology. With the emergence of generative methods and attention mechanisms, this review aims to summarize current advancements and updates in deep learning methods. Our review introduces a distinctive approach by incorporating generative pretrained transformers (GPT), which previous studies have not extensively utilized. From an application perspective, we address the challenge of incomplete data and broaden our scope to include imaging modalities. We group the methods of interest as follows: Non-generative methods (feed forward neural networks (FNNs), graph convolutional neural networks (GCNs), and autoencoders (AEs)) and generative methods (variational methods, GANs, and generative pretrained transformer (GPT) (see Fig. 1). The methods are further categorized by more specific characteristics, including their specific approaches for multi-modal data integration. These include early, intermediate, and late integration (see Fig. 2). In early integration, features from each modality are concatenated before being treated as a single input to the model, whereas methods utilizing intermediate integration treat the modalities as separate entities while being able to learn inter-modality relationships and generate an integrated model or a shared latent space. On the other hand, late integration involves training a separate model for each modality and then combining the predictions to get a final aggregated result. Finally, we discuss advancements afforded by these deep learning frameworks including those that handle incomplete data and those that go beyond molecular -omics data types to incorporate imaging modalities. All of the methods reviewed in this paper are summarized in Table 1.

**Table 1:** Methods summary

Method	Model architecture	Data source	Modalities handled	Handles incomplete data	Task
MOLI [16]	FNN	GDSC [64], PDX [65], TCGA [66]	somatic mutation, CNV, gene expression	No	drug response prediction
SNN [17]	FNN	TCGA [67]	gene expression, DNA methylation	No	classification
GLUER [18]	FNN	self-generated [18]	single-cell gene expression and epigenomics, spatial transcriptomics	Yes	matching cells across different single-cell data modalities
CapsNetMMD [19]	FNN	TCGA [67]	gene expression, DNA methylation, CNV	No	phenotype-related gene identification
MOMA [20]	FNN	TCGA [67], ROSMAP [68]	gene expression, DNA methylation	No	classification
SALMON [21]	FNN	TCGA [67]	gene expression, miRNA expression	No	survival prediction
MiNet [22]	FNN	TCGA [67], KEGG [69], Reactome [70]	gene expression, CNV, DNA methylation, biological pathways	No	survival prediction
DeepOmix [23]	FNN	TCGA [67], KEGG [69], Reactome [70]	genomics, CNV, gene expression, DNA methylation, biological pathways	No	survival prediction
MOGONET [10]	GCN	ROSMAP [68], TCGA [67]	gene expression, miRNA expression, DNA methylation	No	classification
MoGCN [24]	GCN	TCGA [67]	somatic mutation, gene expression, proteomics	No	classification
MOFGCN [25]	GCN	GDSC [64], CCLE [71]	gene expression, CNV, somatic mutation, drug-cell line associations	No	drug response prediction
AGCN [26]	GCN	TCGA [67], STRING [72]	gene expression, CNV, DNA methylation, PPI network	No	classification
DeepMOCCA [27]	GCN	TCGA [67], STRING [72]	somatic mutation, DNA methylation, gene expression, CNV, PPI network	No	survival prediction
Chaudhary et al. [28]	AE	TCGA [67]	gene expression, miRNA expression, DNA methylation	No	dimensionality reduction; clustering
Zhang et al. [29]	AE	TARGET [73], SEQC [74]	gene expression, CNV	No	dimensionality reduction; clustering
Song et al. [30]	AE	TCGA [67]	DNA methylation, gene expression, miRNA expression	No	dimensionality reduction; clustering
DeepAutoGlioma [31]	AE	TCGA [67]	gene expression, DNA methylation	No	dimensionality reduction; clustering
DRIM [32]	AE	CCLE [71]	gene expression, CNV, DNA methylation, genomics	No	drug response prediction and analysis
AE+consensus learning [33]	AE	TCGA [67]	gene expression, DNA methylation, miRNA expression, CNV	No	survival prediction
concatAE/crossAE [34]	AE	TCGA [67]	gene expression, DNA methylation, miRNA expression, CNV	No	survival prediction
MOCSS [35]	AE	TCGA [67]	gene expression, miRNA expression, DNA methylation	No	clustering
DLSF [36]	AE	TCGA [67]	gene expression, DNA methylation, miRNA expression	No	clustering
MultiGATAE [37]	AE	TCGA [67]	miRNA expression, gene expression, DNA methylation	No	clustering
MAE [38]	AE	TCGA [67], STRING [72], miRDB [75]	gene expression, miRNA expression, proteomics, DNA methylation, molecular interaction networks,	No	classification
MvNE [39]	Variational	TCGA [67]	gene expression, miRNA expression, DNA methylation	Yes	representation learning; clustering
DCCA [40]	VAE	10X Genomics [76]	paired single-cell transcriptomics and single-cell epigenomics	Yes	representation learning; clustering; regulatory inference
Multigrate [41]	VAE	10X Genomics [76]	single-cell transcriptomics, single-cell epigenomics, single-cell proteomics	Yes	multimodal data integration and imputation; multimodal atlas construction
GLUE [42]	VAE	SNARE-seq [77], SHARE-seq [78], 10X Multiome [76], Nephron [79], MOp [80]	scRNA-seq, snmC-seq, scATAC-seq	Yes	multi-modal data integration; regulatory inference; multi-omics atlas construction
LSTM-VAE [43]	VAE	Lau et al. [81]	temporal proteomics and metabolomics	No	clustering
CVAE [44]	VAE	TCGA [67]	mRNA, DNA methylation, microRNA	No	classification
OmiVAE [45]	VAE	TCGA [67]	gene expression, DNA methylation	No	classification
MMD-VAE [46]	VAE	TCGA [67]	CNV, mRNA, RNAseq, DNA methylation	No	clustering; classification; survival analysis
OmiEmbed [47]	VAE	TCGA [67], TARGET [73]	gene expression, DNA methylation, miRNA expression	No	multi-task: classification, regression, survival prediction
DeepIMV [48]	Variational (encoder only)	TCGA [67], CCLE [82]	mRNA expressions, DNA methylation, microRNA expressions, reverse phase protein array, DNA copy number, metabolites	Yes	classification
Subtype-GAN [49]	GAN	TCGA [67]	CNA, mRNA, miRNA, DNA methylation	No	clustering
omicsGAN [50]	GAN	TCGA [67], TargetScan Human [83]	mRNA expression, miRNA expression, miRNA-mRNA interaction network	No	classification
CLUE [51]	VAE+GAN	NeurIPS2021 [84], SHARE-seq [78]	single-cell gene expression, single-cell proteomics, single-cell epigenomics	Yes	multimodal single-cell integration
scGPT [52]	GPT	10X Genomics [76], CELLxGENE [85]	single-cell transcriptomics, single-cell epigenomics, single-cell proteomics	Yes	foundation model; diverse tasks: cell type annotation, multi-batch integration, multi-omic integration, perturbation response prediction, gene network inference
Carrillo-Perez et al. [53]	CNN+SVM + late fusion	TCGA [67]	pathomics, gene expression, miRNA expression, CNV, DNA Methylation	No	classification
Chen et al. [54]	AE + late fusion	TCIA NSCLC [86]	radiomics, gene expression, clinical	No	survival prediction
Pathomic Fusion [55]	CNN+FNN+ attention	TCGA [67]	pathomics, genomics, CNV, gene expression	No	survival prediction
Shirkavand et al. [56]	transformer + GAN	ADNI [87], ADSP [88]	radiomics, genomics	Yes	regression; classification
JSRL [57]	GAN	CLAS [89], ADNI [87], AIBL [90]	radiomics (MRI+PET)	Yes	classification
Tulder and Bruijne [58]	CNN	OAI [91], BRATS [92]	radiomics (multiple MRI modalities)	Yes	classification
Morar et al. [59]	FNN	ADNI [87]	radiomics (MRI, PET), biospecimen (CSF), cognitive scores	No	regression
MildInt [60]	RNN	ADNI [87]	radiomics, longitudinal cognitive scores, biospecimen (CSF)	No	classification
Xu et al. [61]	RNN	ADNI [87]	longitudinal radiomics (MRI, PET), demographics	Yes	regression; trajectory prediction
MCNetWang et al. [62]	RNN	ADNI [87], OASIS-3 [93]	longitudinal radiomics (MRI, PET)	Yes	classification
LSN [63]	FNN	ADNI [87], AIBL [90]	longitudinal radiomics (MRI), genetics, clinical	No	classification

*CNV* copy number variation, *CNA* copy number alteration

## Non-generative methods

The first category of methods we will review are non-generative methods. As opposed to generative methods, non-generative methods learn a mapping from the input *X* to the outcome *Y* without modeling the underlying data distribution. In doing so, they focus on the conditional probability distribution of the outcome given the input, i.e., *P*(*Y*|*X*), as opposed to the joint probability distribution of the input and the labels, *P*(*X*, *Y*). Although these methods do not explicitly model the data distribution, and thus do not have the advantages associated with this, their approach is simpler, requiring fewer parameters, and they tend to be less computationally intensive than their generative counterparts. They have been successfully applied to a variety of tasks in multi-omics integration. We organize the non-generative methods in this review into the following categories: (1) feedforward neural networks, (2) graph convolutional neural networks, and (3) autoencoders.

### Feedforward neural networks to integrate multi-omics

The first set of non-generative methods we will discuss is feedforward neural networks (FNNs) which have been adapted to take multiple modalities as input. These range from (1) methods that learn representations separately for modality before concatenating them to produce a final integrated representation, to (2) methods that model inter-modality relationships when constructing a joint representation, and finally (3) methods that additionally consider the biological underpinnings of the modalities by either designing their model architectures to mimic biological organization or incorporating prior domain knowledge.

Sharifi-Noghabi et al. [16] propose MOLI, a late integration deep learning method, by using modality-specific encoding FNNs to learn features separately for each modality before concatenating them into a single multi-omic representation. This concatenated representation is then used as input to a classification sub-network to predict drug response. While this is relatively simple and allows the model to consider the unique distribution of each modality, it may ignore the interactions between modalities.

**Inter-modality Interactions**. To address inter-modality interactions, other methods have been developed to learn features while considering multiple modalities. Bica et al. [17] use a superlayered neural network (SNN), consisting of separate FNN superlayers for each modality as well as cross-connections between them to allow information to flow between the modalities and learn interactions between them.

Another approach called GLUER is introduced by Peng et al. [18] to integrate single-cell multi-omics data and multiplexed molecular imaging assays to match cells across different data modalities for downstream analyses. It first uses nonnegative matrix factorization to derive factor loading matrices that represent common factors shared across modalities, then uses a mutual nearest neighbor algorithm to map many-to-many relationships among cells in different data sets, and finally a deep neural network to project data from different biological assays onto a common feature space while capturing nonlinear relationships between modalities.

Peng et al. [19] approach the learning of inter-modality relationships differently by using a capsule neural network to perform convolution between modalities and samples for a given gene with the goal of identifying novel breast cancer-related genes. This allows the initial feature matrix of each gene to be converted to higher-level and more abstract local features incorporating all modalities.

Finally, Moon and Lee [20] combine multi-omics data using a geometrical deep learning approach by vectorizing and grouping the omics data into modules via a fully connected layer, and then using an attention mechanism to weight the modules based on their relevance for disease prediction. The combination of different omics data into multiple modules allows the model to learn different relationships between the modalities. Furthermore, the method can relate each omics data type to their associated genes, which can then be used to interpret the modules. The most relevant modules for a given phenotype can then be identified using the attention matrix.

**Biological Interpretability**. Another group of methods go even further to allow biological interpretability by either aggregating the data in biologically meaningful ways or incorporating prior domain knowledge. For example, SALMON [21] seeks to use mRNA-seq and miRNA-seq data to predict Cox regression survival in breast cancer by first performing gene co-expression analysis to derive eigengene modules which reduce the dimension of the original feature space into biologically meaningful latent features. Then, these eigengene matrices are input to separate hidden layers in the NN before being combined with copy number burden, tumor mutation burden, demographic and clinical covariates in the Cox proportional hazards regression network. This method enables biological interpretation at the level of co-expression modules rather than individual genes, highlighting potential biological pathways important for breast cancer survival.

Two other methods, MiNet [22] and DeepOmix [23], explicitly incorporate prior biological knowledge. MiNet uses a NN structure that follows a biological system, with a multi-omics layer, followed by a gene layer connecting the multi-omics features to their associated genes, and finally a pathway layer connecting the genes in the gene layer to their corresponding known pathways. These hidden layers represent the hierarchical representations of multiple pathways, and a final hidden node models the interaction effects between pathways, before being input to a Cox layer for cancer survival prediction. Thus, this method captures the interactions between multi-omics data in a manner that reflects true biological organization and is interpretable due to its use of known omics to gene and gene to pathway mappings.

Similarly, DeepOmix [23] is a DNN (deep neural network) including an input gene layer, which takes multi-omics data at the gene-level, and a functional module layer, which utilizes prior biological knowledge to create edges between this layer and the input gene layer that reflect true functional relationships. Each node in the functional module layer is a nonlinear function of different -omics data of the genes it contains. Extracting significant modules corresponding to the prediction result enables interpretation and identification of potential underlying mechanisms of the disease of interest. Thus, allowing for interactions between modalities based on prior biological knowledge allows for more realistic representation of the underlying biological processes and enhances the interpretability of the model.

Based on the methods reviewed in this section, we can see that FNN-based methods are most suited to handle tabular molecular -omics modalities, including gene expression, DNA methylation, miRNA expression, mutation, and CNV. Additionally, FNNs are capable of handling tabular imaging-derived features such as ROI measurements [55, 59, 63] (see Incorporating imaging modalities section). Additionally, some methods can utilize known biological networks to inform their architectures - for these methods, it is ideal that this information is available. Notably, all but one of these methods require all modalities to be measured for every sample. Only GLUER handles incomplete data, as its main goal is to match cells in which different data modalities were measured. However, the FNN in GLUER was used to map different modalities onto a common feature space that had been previously derived using nonnegative matrix factorization, rather than to derive the feature space itself. FNN-based methods make use of all three integration approaches: early, intermediate, and late. Early and late integration strategies do not exploit inter-modality relationships, which is a limitation of these methods. Additionally, the FNN-based methods generally do not handle incomplete data and are limited to tabular -omics data. On the other hand, many of these methods do take into account inter-modality intereactions via intermediate integration. Furthermore, FNNs are simple relative to the other deep learning approaches in this review, and their architectures can be designed to recapitulate biological structure for better interpretability.

### Graph convolutional neural networks

Although several of the FNNs in the previous section attempt to share information between modalities in order to learn inter-modality interactions, they may not fully exploit the correlations between samples [10]. Another set of methods based on graph convolutional neural networks (GCNs) have been developed to more effectively take advantage of both the omics features and the correlations between samples or data types through the use of similarity networks. These similarity networks impose biologically meaningful structure on the model and thus have the advantage of being more interpretable. They also provide a mechanism for incorporating prior biological knowledge, such as interaction networks, into the model. We organize the GCN-based methods reviewed in this paper by how they utilize the graph structure: (1) to incorporate patient similarity network information, or (2) to integrate external biological network information.

**Data-Driven Connectivity**. Some methods generate a patient similarity network (PSN) as part of the GCN in order to take advantage of relationships between samples. For example, Wang et al. [10] introduce MOGONET, a method designed to exploit both multi-omics features and the correlations among samples for biomedical classification tasks. It uses a late-integration approach by first constructing a patient similarity network from each omics data type and then using them to train modality-specific GCNs on the classification task to get initial predictions. It then uses these initial predictions as input to a View Correlated Discovery Network (VCDN) to explore the cross-omics correlations in the label space and generate a final label prediction.

Li et al. [24] also utilize patient similarity information in their method, MoGCN, but take an intermediate integration approach by integrating the modalities before performing classification. They use an autoencoder (AE) to integrate the modalities into a single representation by using multiple encoders and decoders that share the same layer. Similarity network fusion (SNF) was used to construct separate patient similarity networks for each modality before fusing them into a single network. Finally, a GCN takes the patient similarity network and the features of each node output by the AE as inputs for the final prediction. The use of the patient similarity matrix was also beneficial for interpretability: visualizing the PSN provides an intuitive explanation for the clinical diagnosis of a given patient.

**Knowledge-guided Connectivity**. Other methods take advantage of the similarity between biological network structures and graph topology to infuse prior knowledge into the GCN. Peng et al. [25] introduce MOFGCN, which constructs a heterogeneous network utilizing a cell line similarity network, drug similarity network and known drug-cell line associations in order to predict drug response in cell lines. In a similar manner to some methods that construct a patient similarity matrix, they construct the cell line similarity matrix by computing similarity between cell lines for each modality to produce a separate kernel matrix for each data type and then taking the average of the modality-specific matrices to obtain the similarity fusion matrix. Drug similarity is based on their substructure fingerprints. Finally, known drug-cell line associations were incorporated into the model as edges between drugs and cells to help the model learn associations between drugs and cell lines based on their attributes. Drug response was then predicted by reconstructing the cell line-drug association matrix from GCN-derived features.

Another method, proposed by Guo et al. [94] utilizes an attention-based GCN (AGCN) to integrate multi-omics data and prior knowledge from a protein-protein interaction (PPI) network for breast cancer molecular subtype classification. It uses the PPI information to construct a graph with genes as its nodes, where each node is associated with a set of multi-omics features. Associations between data modalities were modeled using two different attention mechanisms. For prediction, the model generates a global graph representation from a global pooling layer and uses this to output predictions for each sample.

Althubaiti et al. [27] also utilize PPIs as background knowledge along with multi-omics data in the context of cancer survival prediction. Their model, DeepMOCCA, integrates germline and somatic variants, methylation, gene expression, and copy number variants using a graph in which nodes represent genes, and edges represent functional interactions between them. They design a set of mapping functions to map the information from the multi-omics data to these nodes. They then use this graph to predict patient survival time using a GCN combined with Cox regression. Besides encouraging biological plausibility in the model, the incorporation of prior knowledge enhances interpretability. Edges between nodes represent functional relationships and may capture dynamic interactions occurring within a cell, as measured by the multi-omics data.

The GCNs covered in this section demonstrate the suitability of these methods for tabular -omics modalities, including gene expression, miRNA expression, DNA methylation, and CNV data, as well as PPI networks for those which incorporate biological knowledge. For the methods that generate cell line or patient similarity networks, having a very large number of cell lines/patients may make the calculation of PSNs very computationally intensive; thus, these methods may only be able to handle a limited number of samples. Furthermore, because of their use of sample similarity information, these methods are most ideal for applications in which structure and similarity among samples is useful. Other limitations of these methods include the fact that none of them handle missing data, although perhaps the use of PSNs could aid in missing data imputation in future approaches. Additionally, the late integration-based approaches may not as effectively learn inter-modality relationships, and even some of the intermediate integration methods simply use SNF or averaging to combine information across modalities rather than learning more complex interactions between them. However, GCN methods have the advantage of better exploiting relationships among samples while integrating multiple modalities, and their network structure is amenable to incorporating biological network information, giving them an advantage over traditional feedforward NNs.

### Autoencoders

Autoencoders (AEs) are another type of non-generative model that have been applied in several methods to integrate multi-omics data. They are commonly used for dimensionality reduction, which is especially useful in dealing with multi-omics data due to the large number of features resulting from combining multiple data types. AEs are useful in learning nonlinear mappings to a low-dimensional latent space. They are typically comprised of two main neural network components: (1) an encoder, which performs the projection to the latent space and (2) a decoder, which projects the latent embedding back to the original space to reconstruct the input data. Two of the important considerations when combining multi-omics data are the principles of (1) consensus, which assumes that model errors are upper-bounded by disagreement between modalities, and (2) complementary, which rules that each modality contains unique information [34]. Using an autoencoder model is advantageous in its ability to account for these properties, and each of the methods reviewed in this paper consider one or both.

**Complementary Learning**. Some methods that are primarily concerned with using AEs for dimensionality reduction for downstream clustering tasks only consider the complementary principle. These methods were developed with the goal of identifying survival-related low-dimensional features that can be used in downstream clustering to determine potential disease subtypes with significant differences in survival [28–31]. Their approach is to concatenate the data across the modalities, use Cox regression to select an initial set of survival-related features, and then input the selected features to an AE to map these features non-linearly to low-dimensional representations. Cox regression is then used a second time to determine a final set of AE-derived features, which are then used for clustering. Since these methods simply concatenate the features across all data types, they extract any unique information held within each data type (complementary), but they do not enforce similarity between modalities (consensus).

Munquad and Das [31] uses a similar pipeline but goes further to incorporate prior knowledge to integrate gene expression and DNA methylation data using known CpG-gene pairs. The use of prior knowledge linking the modalities based on their common associated genes helps to build consensus among them.

Another method, DRIM [32] uses an autoencoder architecture to combine multi-omics data via late-integration to identify potential drug response mediator genes. Rather than inputting the raw data to an encoder, it first encodes each modality separately via omics-specific encoders, and then it concatenates these features and inputs it to an omics-integration encoder to learn relationships among the modalities. LASSO regression is used to select features associated with drug response, and then the decoder is applied to reconstruct the omics data. The significant genes related to the selected features are chosen as potential mediator genes. Thus, DRIM only considers complementary information, but it incorporates prior knowledge linking the multiple omics layers to their associated genes.

**Consensus Learning**. Another method has been developed to only handle the consensus principle. Tong et al. [33] developed an AE with consensus learning to implicitly model the interactions among the modalities by maximizing their agreement. They do this by introducing a consensus regularization to minimize the difference between hidden features learned by each modality, thus integrating the multi-omics data into a common latent space. This method is useful in that it can detect and account for relationships among data types that may reflect biological pathways without having to explicitly model every possible interaction. However, emphasis on maximizing the agreement between modalities without considering the complementary principle may also mean that it does not fully exploit the modality-specific information that is available.

**Complementary and Consensus Learning**. Tong et al. [34] considered both principles when developing concatAE and crossAE, which are separate models designed to handle the complementary and consensus learning, respectively. ConcatAE trains an independent AE with separate reconstruction loss for each modality, then concatenates the features output by each AE for the downstream task-specific model. This allows each of the modalities to have separate influence on the prediction. On the other hand, CrossAE uses the hidden features from each modality to reconstruct the features of every other modality using cross-modality reconstruction loss, which aims to maximize similarity between the latent space representations of every modality. The final representation is the average of the latent space representations from each of the modalities. Although the authors consider both principles, they do not propose a model that accounts for both principles simultaneously.

Some methods have been developed to handle both complementary and consensus principles. Chen et al. [35] developed MOCSS, a method that learns both shared and specific information from multi-omics data for clustering and cancer subtyping. To do this, it applies two autoencoders to extract shared and specific information. Then, it uses an orthogonality constraint to separate the shared and specific information, in addition to contrastive learning on the representations encoded by the shared information autoencoder to align the shared information and enforce consistency between different omics data. Then, a unified representation is derived using both the shared information and specific information representations.

**Similarity Learning**. Other methods handle the consensus principle by extracting and utilizing similarity information from the data, while also incorporating modality-specific information. For example, Zhang et al. [36] propose DLSF, a deep latent space fusion method using a deep cycle autoencoder to learn robust latent representations for each modality, followed by a shared self-expression layer to integrate all modalities by learning a consistent sample manifold. The self-expression layer learns a matrix representing sample similarity that is consistent across all modalities, and then this matrix is used for clustering to identify subtypes. Thus, by learning representations for each modality first and then combining them in a way that enforces consistency across all data types, DLSF incorporates both the specific and shared information across multiple omics data types.

Zhang et al. [37] also use a similarity graph in their clustering and subtyping method, MultiGATAE. This method uses multi-omics data to generate separate similarity graphs among samples, followed by similarity network fusion to derive a fused similarity graph. Then, it uses this network along with the multi-omics data as input to a graph AE, which uses both graph attention and omics-level attention to learn an embedding representation. To help encode a given sample, graph attention exploits similar samples, whereas omics-level attention helps to aggregate the output across modalities while considering inter-modality relationships. The representation is then learned to reconstruct the original similarity graph and then used as the input for clustering.

Ma and Zhang [38] took another approach to incorporate network information into their model: rather than directly encode similarity networks into their model, as is done in graph-based models, they incorporate both domain knowledge and patient similarity networks as constraints. Their proposed method, multi-view factorization autoencoder (MAE), uses separate encoders for each modality as well as a submodule that combines individual views. It uses a linear decoder on which it imposes graph biological knowledge constraints, as well as the fused patient similarity network to constrain the latent representations to be consistent across modalities, thus enforcing consensus. The final representations are derived by taking the sum of the representations from the view-specific autoencoders. In both MultiGATAE and MAE, the use of both view-specific information as well as patient similarity helps to encode both the specific and shared information across modalities. Furthermore, MAE’s use of prior biological knowledge helps guide the model to capture biologically meaningful relationships.

All of the AE-based methods reviewed in this section were designed to handle vectorized input, and therefore, they are well-suited to handle tabular -omics modalities including gene expression, miRNA expression, DNA methylation and CNV data. As demonstrated by MAE, these methods may also be capable of integrating information from molecular interaction networks. All three integration frameworks are utilized among the AE-based methods, where early and late integration approaches that concatenate features across modalities are useful for the complementarity principle by preserving modality-specific information. Some intermediate integration approaches adhere to the consensus principle by maximizing similarity between latent representations of different modalities, while others incorporate both principles. The ability to impose desired properties such as complementarity and consensus on the latent representation is one of the advantages of AEs. Another is that their use of decoders to reconstruct the input helps to ensure that the representations they learn retain the most relevant and discriminative information. This makes them useful for both supervised and unsupervised tasks such as clustering, which was not among the tasks handled by FNNs and GCNs. Among the limitations of these methods is that none of them handle missingness, making them more suited for datasets in which all modalities are measured for each sample. They are also more complex models, consisting of both encoders and decoders, thus increasing their reliance on large sample sizes to sufficiently train their many parameters.

## Generative methods

The next set of methods we will review in this paper are generative. What distinguishes these methods from non-generative methods is that they model not only the distribution of the label space, but also the distribution of the data. That is, generative methods learn the joint probability distribution *P*(*X*, *Y*) of the data *X* and the labels *Y*, whereas non-generative models learn the distribution of the labels conditional on the data, i.e., *P*(*Y*|*X*) [95]. While the approach of non-generative methods is simpler, thus requiring fewer parameters, and focuses on directly solving the problem of mapping inputs to labels, there are also many benefits that can come from modeling the more general distribution of the data. Recent approaches have used generative methods for the application of integrating multi-omics data. Such methods reviewed here encompass variational methods including variational autoencoders (VAE), as well as generative adversarial networks (GANs) and a recently developed generative pretrained transformer (GPT).

### Variational methods

Variational methods model the distribution of the data. This is useful for integrating multi-omics data by learning a single joint latent distribution across all modalities. This joint distribution can then be used to generate a single representation encompassing the comprehensive information contained across multiple omics layers. Explicitly modeling the latent distribution allows for the incorporation of priors that constrain the latent space to have desired properties [96], allowing the resulting embeddings to be meaningful, more robust and generative [43]. This gives variational autoencoders (VAEs) an advantage over the non-variational AEs discussed previously, which tend to have discontinuous latent representations [46] and thus may not be structured in a meaningful manner [43]. Additionally, estimating the latent distribution makes possible the combination of multiple modality-specific embedding spaces into a joint latent space in addition to constraining the individual latent spaces be consistent. Finally, having an estimated latent distribution enables these models to be generative, which can be useful for handling incomplete data. The variational methods reviewed here share the goals of (a) learning biologically meaningful relationships between different omics layers, and (b) dimensionality reduction to overcome the issue of large number of features and small sample size (‘large p, small n’) commonly encountered in multi-omics data. Methods to do this showed two major trends: (1) unsupervised learning to combine multi-omics data into a single integrated representation, and (2) supervised learning to generate representations that contain task-relevant information.

**Unsupervised learning for modeling inter-modality relationships**. Multiple unsupervised methods have been developed with the primary goal of aggregating and learning the relationships between multiple omics modalities for broad downstream analysis. Mitra et al. [39] developed multi-view neighborhood embedding (MvNE) to learn a unified probability distribution of samples across different omics modalities to generate low-dimensional embeddings that preserve the relationships between samples in the new space. They learn probability distributions for each sample for each modality and then combine them using a conflation method to create a single unified distribution. Combining modalities in the probability space circumvents the issue of different data types having incomparable scales.

Zuo et al. [40] take a slightly different approach in their deep cross-omics cycle attention method (DCCA) to jointly profile single cell multi-omics data for multiple downstream analyses. DCCA first encodes each data modality using separate VAEs, and then performs cyclical attention transfer to model the associations between modalities. It also uses a loss function to encourage representations learned in the latent space to be similar to one another while also accurately reconstructing their corresponding original modality. These embeddings were then used for multiple downstream tasks including clustering and visualization, characterizing transcription factor motif activity, and inferring transcriptome regulation from a multi-omics perspective. A limitation of this method is that it requires the modalities to be paired, i.e., the sample from which they were measured should be known. This is often not the case in single-cell data, where the current technologies to simultaneously measure multiple omics are limited and still under development.

**Handling Unpaired Data.** Two other methods, Multigrate [41] and GLUE [42], do handle unpaired single-cell multi-omics data. Multigrate learns a joint representation space that contains information from all modalities. It does this using a product-of-experts (PoE) framework, where the joint posterior is modeled as the product of the conditional marginal posteriors which generate the modality-specific representations. Additionally, it includes a maximum mean discrepancy (MMD) loss to minimize the distance between the joint representations learned by different data sets, thus encouraging consistency even when the data are unpaired. GLUE takes a different approach: it encodes each modality using separate VAEs to learn low-dimensional cell embeddings from each omics type. Rather than use PoE to generate a joint embedding, however, it uses a graph containing prior knowledge of regulatory interactions to associate different omics features to link the omics-specific embedding spaces: a separate graph VAE learns feature embeddings from the prior knowledge graph, and these embeddings are combined with the modality-specific embeddings to integrate them into a common space. GLUE additionally uses adversarial learning to align the cell embeddings of different omics data types. Both Multigrate and GLUE apply their learned representations to constructing multi-modal reference atlases, contributing to an improved understanding of inter-omics relationships.

**Unsupervised learning for dimensionality reduction**. Other unsupervised methods primarily sought to learn low-dimensional latent features to ameliorate the ‘large p, small n’ issue in specific downstream tasks. These include methods developed by Chung et al. [43] and Albaradei et al. [44]. In order to integrate temporal proteomics and metabolomics data, Chung et al. [43] develop a long short-term memory (LSTM)-based VAE architecture (LSTM-VAE) as a dimensionality reduction approach to extract temporal trends in each omics data type. The resulting features were clustered to identify groups of proteins and metabolites that are potentially involved in shared biological pathways during cardiac remodeling. Similarly, Albaradei et al. [44] use a convolutional VAE (CVAE) to extract features from pan-cancer multi-modal data by first concatenating across modalities and then inputting them into two convolutional layers to extract local patterns via sliding filters. These features were then fed into a separate deep neural network to classify tumors as metastatic versus primary. Notably, both of these methods use the VAE to learn low-dimensional representations of the original data in an unsupervised manner (via reconstruction loss), but do not further train these representations for a specific downstream task.

**Supervised learning for generating task-relevant embeddings**. While the unsupervised representation learning methods reviewed above have shown success in downstream tasks, it is possible that when the data labels are imbalanced, or when the primary source of variation in the data is not correlated with the labels being predicted in the downstream task, unsupervised dimensionality reduction may discard critical information [46]. To combat this, many supervised variational methods have been developed to learn low-dimensional representations that are task-oriented.

Zhang et al. [45] developed OmiVAE, which combines a VAE with a classification network that is learned end-to-end. It first concatenates the data across modalities and inputs it to a VAE to extract low-dimensional features. Then, the output of the encoder is connected to a classification network that encourages the network to learn latent representations that contain information relevant to identifying cancer and classifying tumor types.

Hira et al. [46] developed a model, Maximum Mean Discrepancy VAE (MMD-VAE), which has the same structure as OmiVAE except that it uses MMD loss instead of KL-divergence to measure the difference between the posterior and prior latent distributions. This new loss function, which requires all moments of the two distributions to be the same, was proposed to address the issues of uninformative latent features and overestimation of the variance in the feature space which may arise when the traditional ELBO-based loss function is used.

Another method, OmiEmbed [47] consists of both deep embedding VAE networks and downstream task networks, but it extends the task-specific component to multi-task learning. In doing so, it shares information among a diverse set of tasks to obtain embeddings that are adapted to multiple supervised learning problems.

Going beyond the architecture of VAE embedding and supervised learning networks, DeepIMV [48] consists of four main components: (1) modality-specific encoders, (2) PoE to combine the modality-specific latent representations into a joint representation, (3) a multi-view predictor to generate a prediction based on the joint predictions, (3) and modality-specific predictors. It uses an information bottleneck (IB) approach to preserve the most relevant task-specific information from both the modality-specific and joint representations. In doing so, DeepIMV considers both the consensus and complementary nature of the multi-omics data.

From methods reviewed here, we can see that while unsupervised methods have the advantage of not requiring specific labels and learning the inherent structure in the data, supervised methods can be beneficial for extracting specific predictive information, either for a single task or multiple tasks.

Overall, the VAE-based methods in this section were all developed for tabular multi-omics data including gene expression, miRNA expression, DNA methylation, and CNV data. Additionally, some of these methods are capable of handling incomplete data and thus can be applied to paired or unpaired single-cell multi-omic data. Thus, one of the advantages of VAE-based methods is that modeling the latent distribution enables the use of techniques that can infer multimodal information from modalities that are available while allowing for missingness. PoE generates joint distribution using the modalities that are present, and GLUE can link omics-specific embedding spaces from unpaired data using prior knowledge. The generative nature of these methods could also potentially be used to generate one modality from the representation of another. Additionally, modeling the latent distribution enables methods to encourage properties such as adherence to the prior latent distribution and consistency and between the distributions corresponding to different modalities. While most of the methods utilize an early or intermediate integration approach, DeepIMV uses both intermediate and late integration to produce both modality-specific and multimodal predictions, which was shown to be beneficial for classification tasks. Despite these advantages, VAEs are more complex, making them more difficult to train. Furthermore, the number of parameters increases greatly with the number of modalities, which poses limitations on the number of data types that can be integrated for a given sample size.

### Generative adversarial networks

Generative adversarial networks (GANs) are another type of method that models the data distribution, but they are learned via an adversarial procedure. This process uses a generative model to capture the data distribution and a discriminative model that is often trained to distinguish artificial data generated from the modeled distribution and real data [97]. Training the model in an adversarial manner improves both the ability of the generative model to produce realistic data and the discriminative model to distinguish real and generated data, ideally resulting in a close fit to the true data distribution.

One GAN-based multi-omics integration method is Subtype-GAN [49], which handles multiple modalities via a multi-input-multi-output network coupled with an adversarial generation network. It first extracts features from each omics data type separately using fully connected layers to capture their distinct distributions, before inputting them to another fully connected layer which generates the parameters of a distribution for the shared latent representation. The decoder is trained to reconstruct each individual modality from the shared representation. In this way, Subtype-GAN follows a VAE-like structure. Also like VAEs, it assumes a prior distribution for the latent variables, which acts as a regularizer to prevent overfitting and ensures the smoothness of the latent space. However, it also trains a discriminator to distinguish samples of the learned shared embedding space from those of the prior distribution. This ensures that the posterior distribution of the shared latent representations matches the prior Gaussian distribution. Finally, the features from the shared layer are used in consensus clustering to identify cancer subtypes. Thus, this method mainly takes advantage of adversarial learning to constrain the shared embedding space to match the prior, but it does not use it to help learn the relationships between different modalities.

On the other hand, omicsGAN [50] directly leverages adversarial learning to learn inter-modality relationships. It is designed to integrate two modalities as well as their interaction network by learning a Wasserstein GAN for each modality to generate updated embeddings that encapsulate information from both omics data types as well as their interactions. Using both the real data from a given modality and the adjacency matrix of their interaction network, the generator is trained to synthesize the other modality. A discriminator is then trained in an adversarial game to differentiate the real and synthetic data. The resulting output is taken to be the new feature set for the other modality. Experiments demonstrated that the synthetic data containing information from both modalities and their interaction network performed better than the original data in cancer outcome classification. Another method, CLUE [51], combines the VAE architecture with adversarial learning. It learns inter-modality relationships using both self-encoders and cross-encoders that learn latent representations of each modality from itself and from each of the other modalities, respectively. A discriminator is also trained to distinguish which modality a latent representation is derived from to enforce consensus between latent representations inferred from different modalities.

Therefore, we can see that GAN-based methods use adversarial loss by training a discriminator to distinguish data generated by two different distributions in order to learn representations that better recapitulate some desired distribution. This type of learning can be leveraged for the purpose of regularization, as was done in Subtype-GAN, or to help ensure consensus between different data types, as was done in omicsGAN.

As with all other method types reviewed thus far, the GAN methods in this section were designed for tabular -omics data, including gene expression, miRNA expression, and CNV. Additionally, CLUE can handle unpaired single-cell -omics modalities, and omicsGAN requires an interaction network. Other GANs have been applied to imaging data such as MRI and PET [57] (see Incorporating imaging modalities section). Although omicsGAN requires complete data for training, its ability to generate synthetic data for each modality using the other modality suggests that it could potentially be applied to incomplete data at test time. All methods in this section use the intermediate integration approach, in which they generate representations of each modality while also learning the relationships between data types to encourage their agreement. As we have seen, the adversarial learning strategy utilized by GANs allows them to enforce consensus among latent distributions corresponding to different modalities and can also serve as a regularization mechanism. Despite these advantages, GANs are more complex, requiring the training of both generators and discriminators, and thus they are limited in the number of modalities they can handle while requiring a large sample size.

### Generative pretrained transformer

A rapidly growing area in artificial intelligence is the development of foundation models such as generative pretrained transformers (GPTs) which are trained on vast data sets to learn the general patterns inherent in the data before being fine-tuned for specific tasks. Taken from the field of natural language processing (NLP), these models are now being adapted to other disciplines, including the biomedical domain. For NLP applications, the transformer architecture [98] has enabled representation learning from sentences by using an attention mechanism that can relate elements between any two locations of arbitrary distance in the sequence. Multi-head attention enables the learning of multiple such relationships. Additionally, the transformer architecture is amenable to parallelization, increasing its computational efficiency. For application to biomedical data, the transformer is still useful for relating components that make up a larger biological entity, such as genes in a cell. In the case of multi-omics integration, the attention mechanism can also be used to capture relationships between different modalities.

Recently, Cui et al. [52] proposed scGPT, a foundation model for single-cell omics data. Whereas NLP-based models are trained to model text composed of words, scGPT models cells composed of genes and their protein products. Through pre-training on large-scale non-sequential single-cell omics datasets composed of over 33 million cells, it learns cell and gene representations simultaneously, which capture the general biological patterns and interactions in single-cell data. It can then be fine-tuned for specific tasks, including multi-omics integration. Its architecture consists of stacked transformer blocks with specialized attention masks [98] for generative pretraining via self-supervised learning. The input layers include gene tokens, expression values and condition tokens, which can represent attributes such as modality, batch, and experimental condition. In the case of multi-omics integration, the condition tokens represent the modality from which the features are taken. These tokens are concatenated with the transformer output before being input to task-specific fine-tuning modules. This prevents the transformer from biasing attention to be greater within features of the same modality while underestimating associations with features in different modalities.

In experiments, scGPT achieved state-of-the-art performance in multi-omics integration. Its performance on downstream tasks also improves as the pretraining data size increases, indicating that as more data becomes available, GPTs are likely to become even more powerful, and thus are a promising approach for multi-omics integration, among other tasks.

scGPT was designed for single-cell -omics, including transcriptomics, epigenomics, and proteomics, and thus is limited to these data types. However, other transformer-based methods have been applied to imaging modalities such as radiomics [56] or clinical text [99, 100]. As scGPT is a large foundation model, it requires vast amounts of data for pre-training, and thus, GPT models in general are limited to data types for which such volumes are available. As a result, they are also very computationally expensive to train. On the other hand, pre-training on a vast dataset has been shown to enable better performance on a variety of specific downstream tasks, as scGPT exhibits superior performance on both paired and unpaired datasets.

## Recent advancements and future directions in deep learning for multi-omics integration

Deep learning-based approaches build off of previous statistical methods to integrate multi-omics data by enabling the modeling of complex and nonlinear interactions between data types, as we have seen in this review. In some of these methods as well as additional approaches, we see emerging themes that point toward future directions in the field of multi-omics integration. These include the ability to handle incomplete multi-omics data as well as going beyond the use of molecular omics to utilize imaging-based omics.

### Handling incomplete multi-omics data

A common challenge in analyzing multi-omics data is that samples are often missing one or more modalities. Many multi-omics integration methods either exclude samples that are missing any modalities or impute missing values as a data preprocessing step. The former results in a reduced sample size while not allowing the full usage of all information contained in the dataset, and the samples with missing values may not be a random subset of the data [101]. The latter may bias the relationships between features toward similarities in imputation, potentially negatively impacting downstream analyses [101]. To combat this, some of the deep learning methods in this review have been developed to handle incomplete multi-modal data within their frameworks. Each method reviewed here takes one of following strategies: (1) learning a joint probability distribution from the available modalities, (2) cross-learning, and (3) combining unpaired single-cell multi-omics data using inter-modality relationships.

DeepIMV [48], Multigrate [41], and MvNE [39] learn joint probability distributions of the latent variables that can handle the case of missing modalities. Both DeepIMV and Multigrate take the product-of-experts approach, which represents the joint latent distribution as the product of the single-modality latent distributions. When a specific modality is missing, the joint distribution can still be determined from the modalities that are present while ignoring missing modalities. Thus, this enables the generation of a joint embedding regardless of a sample’s modality-missing pattern. DeepIMV still preserves modality-specific predictive information by training modality-specific predictors as well as the PoE-derived joint representation. Multigrate can even impute missing modalities from the joint representation using its decoder to reconstruct all modalities, even if some were missing in the input. MvNE takes a different approach by modeling and combining probability distributions for each sample for each modality into a unified probability distribution using a conflation method. In the case of incomplete data, this approach simply changes the definition of unified probability based on the modality-missingness pattern.

Other methods use cross-learning approaches that enable the inference of one modality’s representation from another. In addition to using self-encoders that generate a latent representation from each modality, CLUE [51] uses cross-encoders which enables all modalities to generate latent representations for every other modality. When a given modality is missing, its latent representation can still be inferred from the available modalities. DCCA [40] can also handle missing modalities by generating missing omics from the omics that are present. This is because it uses cyclical attention transfer to minimize the distance between latent features corresponding to different modalities. For a given modality, it learns representations of each omics data that has similar embeddings with and accurate reconstruction of that modality. In experiments, scATAC-seq data generated from scRNA-seq data was positively correlated with the true scATAC-seq data 100% of the time, and correlations were 0.9 and above on two different data sets.

Moreover, methods designed for unpaired data handle incomplete data by default. GLUER [18] and GLUE [42] both use the relationships between different omics modalities to generate representations that share information across them. GLUER projects all datasets onto a subspace with shared structure, then identifies pairs of similar cells in that space based on the mutual nearest neighbor algorithm. Based on these cell pairs, it then learns nonlinear mapping functions between modalities using a deep neural network. Finally, it computes a co-embedded data matrix using these nonlinear mapping functions which can be used for data imputation. GLUE learns new feature embeddings that link all omics data by incorporating both omics dataset-specific encoders and information derived from a guidance graph of prior knowledge about regulatory interactions.

Finally, single-cell multi-omics data analysis often encounters challenges due to sparsity, where not every cell expresses each gene. To address this, the typical approach involves two key steps: first, dimension reduction techniques are applied to simplify the data while preserving important features. Second, advanced integration methods, possibly including machine learning or neural networks, are used to combine data from different modalities. This helps to learn from different modalities to impute or fill in missing data, utilizing the strengths of diverse data types to enhance the analysis. GLUER [18] effectively handles sparsity in single-cell -omics and imaging data by leveraging Nonnegative Matrix Factorization (NMF) to learn lower-dimensional shared factors among diverse data sets while enhancing data interpretability. GLUE [42] applied linear dimensionality reduction as the first transformation layers in the encoder in addition to utilizing a guidance graph encoding regulatory interactions to link the different -omics data types. CLUE[51] uses modality-specific encoders and cross-encoders trained on partially paired single-cell multi-omics data to be able to generate lower-dimensional integrative representations from sparse, modality-incomplete data.

The ability to leverage all samples in a dataset regardless of their patterns of missing modalities is an important characteristic of multi-omics integration methods. The methods reviewed here demonstrate some current approaches that have been developed to handle this, and it is expected that we will see even more development in this area as the field advances.

### Incorporating imaging modalities

Additional deep learning methods go beyond the multi-omics data discussed earlier in this review to analyze imaging modalities. There are many clinical settings where images are available, including pathomics (e.g. histology slides) and radiomics (e.g. magnetic resonance imaging (MRI), positron emission tomography (PET), and computed tomography (CT)). These data contain rich visual information that can be leveraged for predictive insights. For example, Lu et al. [102] used a U-net-based convolutional neural network to identify and extract cell-level lymphocytic regions in H&E-stained images and found that spatial features derived from these regions had strong associations with gene expression and somatic mutations and were also predictive of patient outcomes. Notably, when raw images are used as inputs, convolutional neural network (CNN) architectures are often utilized to process them. CNNs have the same basic structure as FNNs, but they additionally use convolutional layers consisting of filters that perform downsampling by aggregating local regions of the image before vectorization. Thus, the representations learned using convolutional filters preserve spatial contextual information not captured by the molecular multi-omics modalities discussed earlier in this review. To take advantage of the available visual information provided by pathomics and radiomics data, several deep learning methods have been developed that (1) extract imaging and multi-omics features separately before combining them via late fusion for downstream tasks, (2) extract both imaging and multi-omics while modeling interactions between modalities, (3) utilize multiple imaging modalities, and (4) utilize longitudinal multi-modal imaging data.

A commonly used approach to integrating imaging with multi-omics data has been via “late fusion”, in which features from each modality are extracted separately before being combined for downstream tasks. Deep learning methods are often important for extracting features from images or deriving latent features from multi-omics data. To classify non-small-cell lung cancer (NSCLC) subjects, Carrillo-Perez et al. [53] fused whole-side imaging with RNA-seq, miRNA-seq, copy number variation, and DNA methylation data by training an independent machine learning model for each modality: they used a CNN for feature extraction and prediction for the histology images, and SVMs for the molecular data modalities. They then fused the probabilities via weight-sum optimization to obtain a final prediction. Similarly, Chen et al. [54] combined CT images, gene expression, and clinical factors to predict survival in NSCLC patients. They manually extracted features from the segmented CT images and used an autoencoder framework to learn latent features from the gene expression data. Then, they calculated risk scores from each modality separately and fused them for the final prognosis prediction. While the late fusion approach can easily handle inter-modality differences by using separate feature extraction models for each data type, it ignores interactions between modalities.

Other methods do account for these interactions: GLUER [18], which we have previously discussed in this review, is designed to handle multiplexed molecular imaging data as well as multi-omics, treating it as an additional modality that undergoes the same processing alongside the other omics data: joint NMF for identifying common factors shared across data sets, mutual nearest neighbor algorithm for mapping relationships among cells across data sets, and deep learning NNs to capture the nonlinear relationships between datasets. Additionally, Chen et al. [55] present Pathomic Fusion, an end-to-end integrated framework to fuse histology image with mutation, CNV, and RNA-seq features for cancer survival outcome prediction. Features are extracted from each modality separately based on the supervised learning task, and then multimodal fusion is performed using gating-based attention to control the level of influence of each modality on the outcome and the Kronecker product to model pairwise feature interactions between modalities. CNNs or parameter efficient GCNs are used to extract the histology features, and a feed-forward network is used for the genomic features. Other methods have been developed to integrate radiomics and genomic data: Shirkavand et al. [56] propose a framework that utilizes MRI and SNP data to predict cognitive degeneration and disease outcomes in Alzheimer’s disease subjects using a transformer to extract imaging features and a GAN to learn the relationship between MRI and SNP data.

In some domain areas, multiple imaging modalities are available. For example, Alzheimer’s disease patients often have both MRI and PET imaging modalities available. To take advantage of these data, methods have been developed to handle multi-modal imaging data. Liu et al. [57] propose a joint neuroimage synthesis and representation learning (JSRL) framework to predict conversion from subjective cognitive decline (SCD) to MCI using MRI and PET data. It uses a GAN to to handle incomplete data by synthesizing missing PET images and generate multi-modal features, as well as a classification network to fuse the multi-modal features for prediction. Tulder and Bruijne [58] combine multiple MRI sequences using an axial CNN, which is an autoencoder-like model that learns a shared representation across multiple modalities by averaging the representations from each separate modality.

Additional methods have gone even further to leverage longitudinal multi-modal imaging data. Morar et al. [59] use a deep fully connected NN to predict cognitive score at multiple future time points using MRI neuroimaging measurements, cerebral spinal fluid (CSF) measurements, PET measurements, and cognitive scores but do not leverage multiple time points as input to the model. Methods that do take longitudinal input data rely on sequential model architectures such as recurrent neural networks (RNNs). RNNs share the same architecture as FNNs, except they can process sequential data by repeatedly applying the neural network to each element of the sequence one-at-a-time while also considering the output of the previous time point. Lee et al. [60] present MildInt, a method to integrate longitudinal cognitive performance and CSF data, as well as MRI and demographic information using an RNN-based architecture to learn longitudinal feature representations in each modality separately, before concatenating the representations across modalities for final classification. Xu et al. [61] and Wang et al. [62] also utilize RNN-based architectures to model temporal patterns in the longitudinal data and impute missing time points, while using other deep learning techniques to learn cross-modality representations. Bhagwat et al. [63] use longitudinal cognitive scores to model Alzheimer’s disease progression trajectories and then develop a longitudinal Siamese network (LSN) to combine MRI data from two time points, along with genetic and clinical information, to predict the prognostic trajectories of individual subjects. The LSN network consists of two FNNs, each corresponding to multi-modal data from one time point (baseline or follow-up), with weight-sharing branches to combine information across the two times. This allows the LSN to produce an output that is representative of the change in the subject over time (e.g., brain atrophy).

Thus, many deep learning methods have been developed to integrate modalities other than the common molecular omics data seen in the majority of this review paper. As we have seen, images contain rich information that can complement these molecular -omics data types. In medical domains that heavily rely on imaging data, such as cancer and neurodegenerative disease, these modalities are particularly useful. As the field progresses and even more data becomes available, as well as methods that can handle missing modalities, we expect to see further development combining all of these data types to leverage all available information from a given sample for predictive insights.

## Conclusions

In this review, we presented several recent deep learning-based approaches to integrate multi-omics data for various downstream applications. Deep learning methods are valuable to the problem of fusing diverse datasets with complex interactions. We categorized the approaches into two main types: non-generative and generative, where generative methods learn distributions of the data and their latent representations, enabling the use of constraints on the embedding space to impose certain desired properties. Non-generative methods included feedforward neural networks, graph convolutional neural networks, and autoencoders, and generative methods included variational methods, generative adversarial networks, and a recently developed generative pretrained model.

Although all model architectures discussed in this paper are capable of handling tabular data, including multi-omics and imaging derived features, some are better suited for data with missing modalities, including GANs, VAEs, and the GPT. These model types are able to learn inter-modality relationships that allow for either cross-modality or joint representation inference. For methods that incorporate sample similarity or biological interaction information, GCN-based methods may be preferable, as their graph-based architecture matches the network structure of the data. Additionally, convolutional neural networks (CNNs) and transformers are particularly well-suited for handling radiomics, as they are better capable of processing 2D and 3D data via convolutional filters and attention, respectively. Finally, recurrent neural networks (RNNs) are most prominently used for handling longitudinal data, as we have seen in the case of Alzheimer’s disease. The sequential nature of RNNs makes them amenable to future trajectory and prognosis prediction. In order to handle longitudinal and multimodal data, a combination of RNN and VAE-based methods [61, 62] may be ideal to handle both the temporal and cross-modal dimensions.

One of the pervasive issues when handling large and diverse data types is sparsity as well as various patterns of missing modalities. Numerous recent methods have been developed to handle this, most of which are generative due to their ability to produce synthetic data for all modalities from a joint representation. As data incompleteness is a common and important issue, we expect to see more methods that can handle this in the future. Additional methods go beyond the traditional molecular -omics data types (e.g. genomics, transcriptomics, epigenomics) to consider imaging modalities (e.g. pathomics, radiomics). Fewer methods combine both molecular omics and imaging modalities, and we expect to see more development in this area to leverage all available data and provide an even more complete picture of each subject. Being able to comprehensively capture the state of each sample should enable a more nuanced understanding of the biology underlying disease outcomes, improving performance on downstream predictive tasks.

## Data Availability

Not applicable.

## Abbreviations

AE: Autoencoder
CNN: Convolutional neural network
FNN: Feedforward neural network
GAN: Generative adversarial network
GCN: Graph convolutional neural network
GPT: Generative pretrained transformer
LSTM: Long short-term memory
MMD: Maximum mean discrepancy
NN: Neural network
PoE: Product-of-experts
PPI: Protein-protein interaction
SNF: Similarity network fusion
VAE: Variational autoencoder

## Data availability

No datasets were generated or analysed during the current study.
